# Gunshot Injury With Bone Defect of the First Metatarsal Bone: A Presentation of 2 Cases Treated With an Iliac Crest Structural Graft, Internal Fixation, and Bone Morphogenic Protein 2

**DOI:** 10.1177/19386400241278026

**Published:** 2024-09-18

**Authors:** Elisabeth Ellingsen Husebye, Geir Stray Andreassen, Are Haukåen Stødle

**Affiliations:** Division of Orthopaedic Surgery, Oslo University Hospital, Oslo, Norway; Division of Orthopaedic Surgery, Oslo University Hospital, Oslo, Norway; Division of Orthopaedic Surgery, Oslo University Hospital, Oslo, Norway

**Keywords:** gunshot injury, metatarsal defect, structural iliac crest graft, BMP 2, gunshot injury to the foot

## Abstract

Gunshot injuries to the foot with segmental bone defects can be challenging to treat. When the vascularity is intact and the soft tissues allows, the goal should be to reconstruct the bony defect. We present 2 cases of a gunshot injury to the foot with a defect of the first metatarsal bone. Both cases were treated, with favorable outcome, with a structural iliac crest graft, internal fixation, and bone morphogenic protein 2.

**Level of Evidence:** V, cases series, technical

“The degree of tissue trauma following a gunshot depends on the tissues involved, the type of firearm, the projectiles physical characteristics, velocity, and distance of fire.”

## Introduction

Traumatic injuries with segmental bone defects can be difficult to treat. Various techniques are well-described for the treatment of femoral and tibial defects.^1,2^

The literature regarding reconstruction of bone defects in the foot, however, is limited. Case reports describe the use of the Masquelet technique,^3,4^ iliac bone grafting,^5^ nonvascularized,^6^ or vascularized fibula graft.^7,8-10^

Following a gunshot injury of the foot, it can be challenging to achieve soft tissue coverage and optimize the alignment, length, and stability of the foot that also allows for normal function and weightbearing. Changes in foot configuration, instability, or joint destruction may lead to changes in load distribution, impaired function or a foot that will not fit into a normal shoe. Cases with severe soft tissue defects or compromised circulation often warrant partial amputation of the foot. A reconstruction, however, can be an option in cases with intact vascularity and only limited soft tissue injury. The goal of the reconstruction of the injured foot is to return to pain-free ambulation, and the surgical procedures should aim to reconstruct a foot that can withstand the biomechanical demands of the normal gait cycle.

In this report, we present 2 cases of a gunshot injury to the medial aspect of the foot leaving a bone defect of the first metatarsal bone (MT1). Both cases were treated with an iliac crest structural graft, internal fixation, and bone morphogenic protein 2 (BMP-2). Management strategies and patient outcome are presented.

## Patient History, Case 1

**Figure 1.**
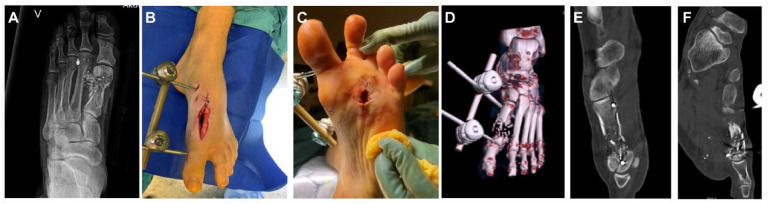
The figure shows the anteroposterior radiograph of the left foot demonstrating the projectile fragments and a shortened fragmented first metatarsal (1A), the left foot with a gunshot injury with a debrided wound dorso medial and central plantar (1B and C). The foot is stabilized with an external fixator, and computed tomography scans of the injury demonstrating the metatarsal defect and metatarso phalangeal joint (1D, E and F).

A healthy, nonsmoking, 36-year-old male had a low-velocity gunshot injury to the left foot caused by a pistol shot with a 9 mm full metal low-energy jacket ammunition at the distance of 1.5 meters. The projectile resulted in fragmentation of the diaphysis of the MT1 with additional fracture lines involving the metatarsal head (Figure 1A).

The projectile penetrated the dorso-medial aspect of the foot at the level of the MT1 and excited plantar to the distal second metatarsal bone (Figure 1B and C).

The neurovascular status was not compromised. Meticulous irrigation and soft tissue debridement was conducted, carefully removing necrotic or nonviable tissues, along with multiple projectile fragments. Repeated debridements and irrigations were performed the following days. A 32-mm segment of the first metatarsal bone, except a thin and fragmented medial cortex, was missing. The metatarsal head was still present (Figure 1D-F). The patient received perioperative intravenous antibiotic prophylaxis with Cefazolin when surgical debridement was performed. An external fixator was applied to obtain stability and length (Figure 1D-F).

**Figure 2.**
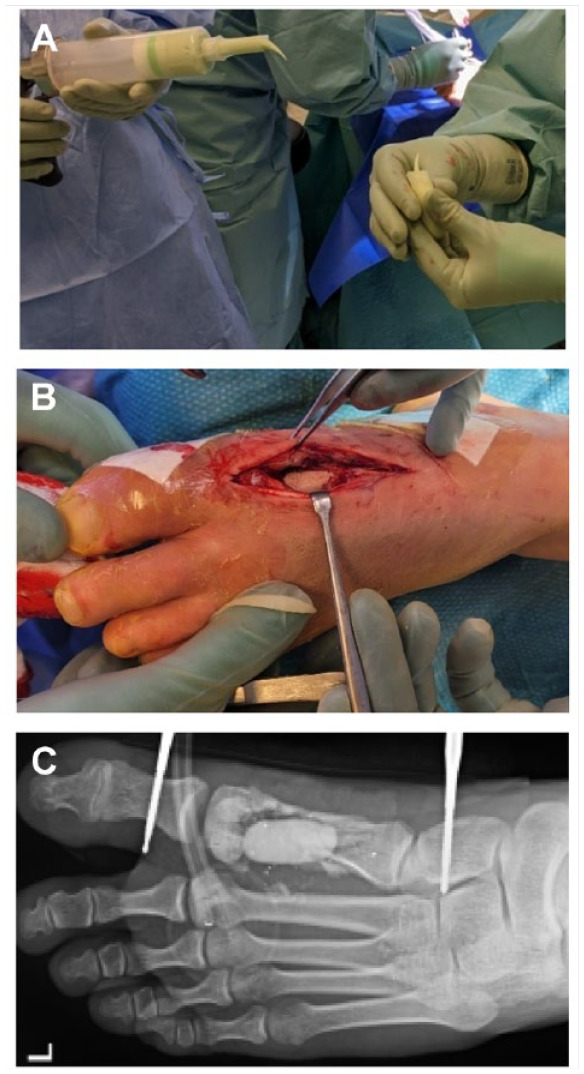
The cement spacer was prepared (2A), and installed into the bony defect (2B). The radiograph shows the cement spacer in the region of the first metatarsal bone (2C).

Ten days post injury, a bone cement spacer (PalacosR +G pro, Heraeus Medical, Wehrheim, Germany) was left in the bony defect for 6 weeks (Figure 2). The external fixator was replaced to avoid the placement in zone of injury.

### Surgical Procedure, Reconstruction

**Figure 3.**
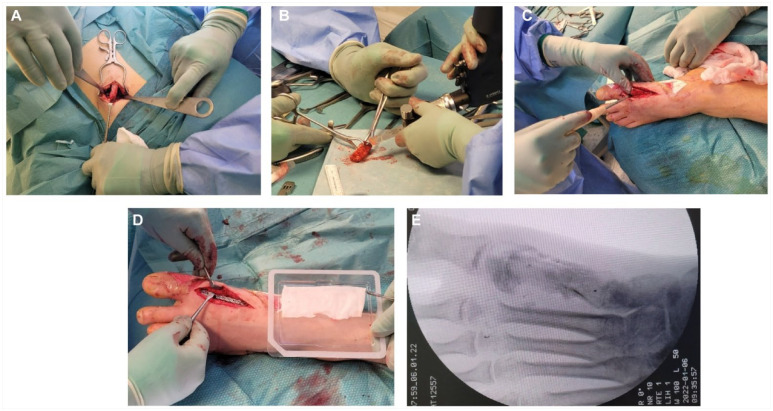
An iliac crest bone graft was harvested from the ipsilateral side (3A), adjusted (3B) and installed into the defect (3C), stabilized with a plate, and finally covered with a sponge soaked with BMP2 (3D). The perioperative radiograph demonstrates the bone graft in the first metatarsal bone prior to plate fixation (3E).

At 6 weeks, reconstruction of the metatarsal defect was performed. The preoperative first metatarso-phalangeal joint (MTPJ-1) range of motion (ROM) was not recorded. The biomembrane encapsulating the cement spacer was carefully opened, and the spacer was removed. The ipsilateral iliac crest was prepared, and a tricortical autograft was harvested with an oscillating saw. The bone graft was adjusted to match the metatarsal defect, and the bone graft measuring 35 × 12 × 16 mm was implanted in the osseous defect with a press-fit technique. A 9-holes stainless steel low profile 2.4 mm T-plate (EVOS MINI Plating System, Smith&Nephew, Mempis, USA) was used to stabilize the metatarsal head fragments, the bone graft and the proximal part of the first metataral bone (Figure 3). A collagen sponge soaked with dibotermin alfa, a recombinant human bone morphogenic protein 2 (BMP-2) (InductOs, Medtronic BioPharma, Heerlen, The Netherlands), was implanted on the dorsal and dorsolateral aspect of the ostheosynthesis. The wound was closed without tension in 2 layers.

### Postoperative Treatment and Follow-Up

**Figure 4.**
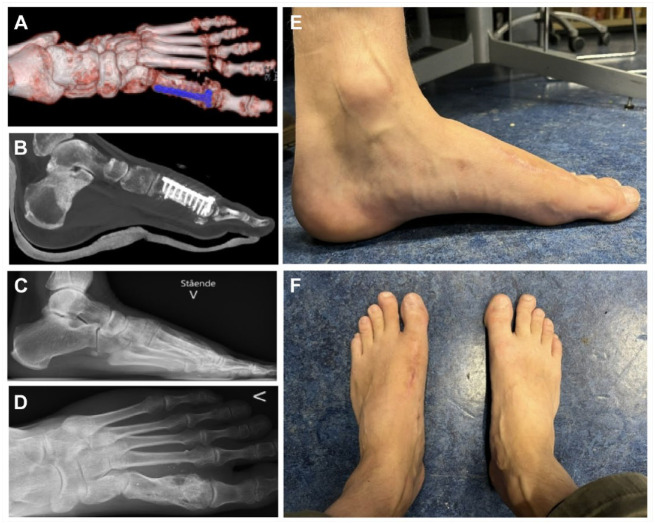
The figure shows the postoperative computed tomography scan, with the bone graft and the metal plate for fixation (4A and B), the lateral and anterioposterior radiographs show the left foot 1 year after iliac graft implantation with integration of the graft (4C and D), the left foot from medial view (4E), and the left and right foot from the anterior view (4F) 23 months after surgery.

A postoperative computed tomography (CT) scan was performed (Figure 4A and B).

The patient suffered from pain in the iliac crest region due to graft harvesting, the first 2 to 3 weeks after the operation. He had no hyperaesthesia/anaestesia related to the lateral cutaneous nerve.

The patient was non-weightbearing in a cast for 6 weeks and partial weightbearing in a Walker boot for another 6 weeks. Computed tomography scans obtained 5 months after surgery showed integration of the bone graft. The patient walked without a limp at 5 months follow-up. Owing to the distal placement of the plate in addition to a slightly displaced dorsal fragment of a metatarsal head fragment, dorsiflexion of the great toe was reduced. The dorsiflexion of the toe improved after removal of the hardware and prominent bone fragment 10 months after graft implantation.

The patient partially returned to work 12 weeks after surgery and returned to a physically demanding full time posistion 12 months after surgery.

The radiographs obtained 12 months postoperatively demonstrated integration of the bone graft, hypertrophy of the metatarsus/graft and moderate narrowing of the joint space of MTPJ-1 (Figure 4C and D). At 23 months follow-up, he reported a visual analogue scale score (VAS) for pain when resting at 0, and a VAS score at 2 when walking. The American Orthopaedic Foot & Ankle Society (AOFAS) midfoot scale score was 82, the Self-Reported Foot and Ankle (SEFAS) score was 41, and the Manchester-Oxford Foot Questionaire (MOxFQ) for walking/standing was 14, for pain 25, and for social interaction 13. He had little complains except that he needed wider shoes than he wore preinjury. The foot is well aligned and the patient describes the result after surgery as very satisfactory (Figure 4E and F).

## Patient History, Case 2

**Figure 5.**
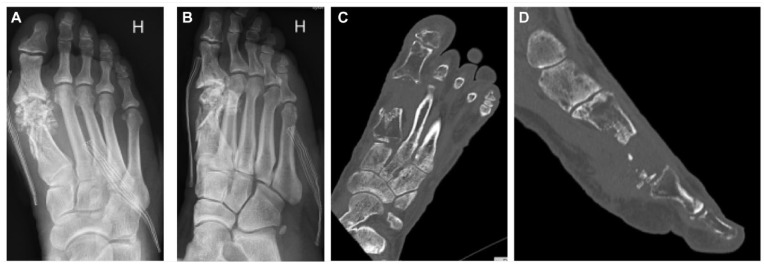
The initial anterioposterior and oblique radiographs show a multifragmented first metatarsal bone, fractured hallux sesamoids and proximal first phalanx (5A and B), and the computed tomography scans show the preoperative state of the injured right foot, with a defect of the first metatarsal bone including the metatarsal head (5C and D).

A nonsmoking 37-year-old male with a previous history of arthritis urica, otherwise healthy, acidentally shot himself with a hunting rifle at short distance, causing a through-and-through injury to his right foot. The projectile penetrated the foot dorsolateral and exited medially at the level of the head of the MT1. The initial radiographs demonstrated multifragmentation of the distal metatarsus including the metatarsal head and the hallux sesamoids. The base of the proximal phalanx was also fractured (Figure 5A and B).

Initial treatment was performed at the local hospital. Wound debridements, irrigation, and removal of nonvital bony fragments and necrotic tissues was performed. The patient received per oral Dicloxacillin for 3 weeks following the injury, but without any information on wound infection or isolated bacteria. The foot was stabilized with an external fixator for 7 weeks. Thereafter, the foot was placed in a Walker boot. The patient had reduced skin sensation of the great toe. The vascular status was not compromized. No cement spacer was used in the bony defect to create a biological membrane. At the time of referral to our department at 15 weeks after injury, the wound was closed and without signs of infection, and a segment of approximately 30 mm of the MT1 was missing, including the metatarsal head. The sesamoids were also mostly missing (Figure 5D and E).

### Surgical Procedure, Reconstruction

**Figure 6.**
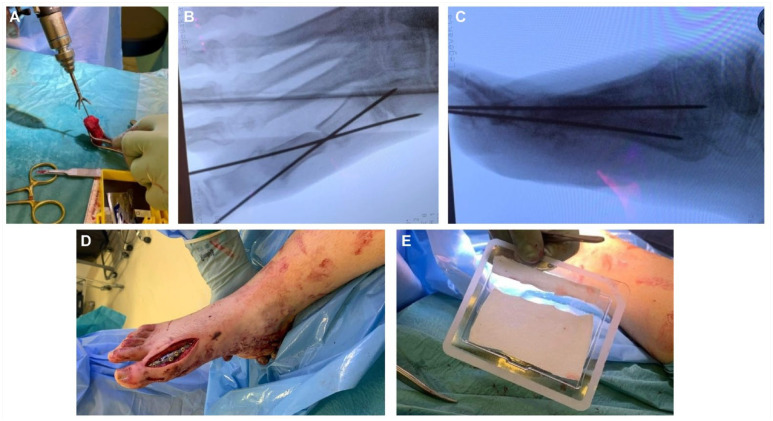
The figure shows the reaming of the graft (6A), the temporary fixation of the graft (6B and C), the stabilization with a plate (6D), and the sponge soaked with BMP-2 (6E).

Tissue samples for bacteria cultivation were obtained after wound irrigation, but no bacteria were isolated. Similar to case 1, the ipsilateral iliac crest was prepared and a tricortical autograft was harvested with an oscillating saw. A 40-mm graft was chosen to allow for shortening of the graft during the adjustment to the proximal phalanx and the proximal first metatarsal bone. The MTPJ-1 was not to be saved due to the missing distal metatarsal fragment, the abcense of most of the hallux sesamoids and the initial fracture of the base of the proximal phalanx. A cup and cone reamer (MTP reamer, Gorilla MTP Plating Systeme, Paragon28, Denver, Colorado, USA) was used for optimal adjustment of the distal part of the graft to the proximal phalanx (Figure 6A). A 5 degrees 9-hole titanic 1.6 mm plate (Gorilla MTP revision plate, Gorilla MTP Plating System, Paragon28, Denver, Colorado, USA) was used for stabilization (Figure 6B-D). Cancellous bone from the iliac graft was packed around the structual graft before a collagen sponge soaked with BMP-2 (InductOs, Medtronic BioPharma, Heerlen, the Netherlands) was implanted on the dorsal aspect of the ostheosynthesis (Figure 6E). The wound was closed in 2 layers without tension.

### Postoperative Treatment and Follow-Up

The patient had pain related to graft harvesting from the iliac crest the first weeks after surgery, but no long-term pain and no injury to the lateral cutaneous nerve at follow-up.

**Figure 7.**
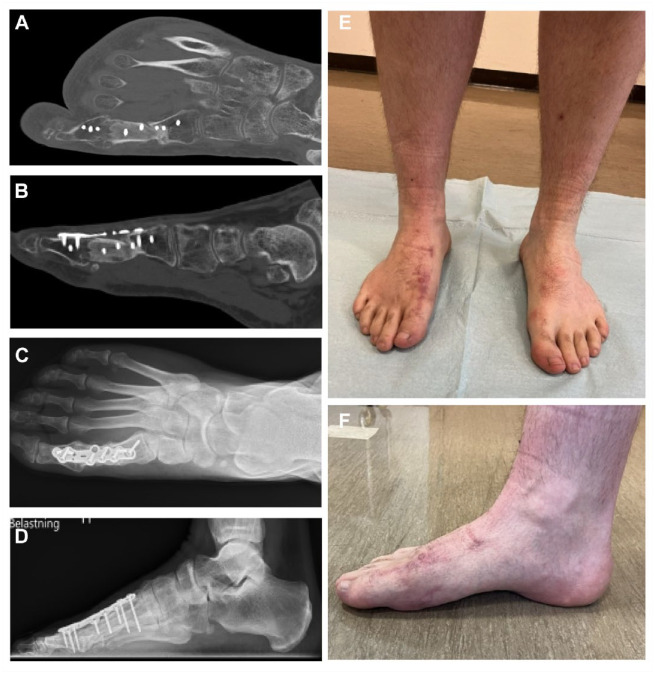
The figure shows the anteroposterior and lateral computed tomographic scans of the right foot 5 months after the graft implantation. Both the distal and the proximal part of the graft seems integrated with the proximal phalanx and the proximal end of the first metatarsal bone, even though the central part of the graft shows hyperdensity (7A and B). The anteroposterior and lateral radiographs at 16 months after graft implantation show a continuity of the first metatarsus and a fused metatarsophalangeal joint (7C and D). Figure 7E shows the anterior view of the right and left foot and Figure 7F the medial view of the right foot at 16 months after surgery.

The patient was nonweightbearing for 8 weeks, and partial weightbearing in a Walker boot for another 4 weeks. Computed tomography scans obtained 3 months after surgery showed complete integration of the bone graft distally, but not proximally. Therefore, the Walker boot was continued with partial weight bearing for another 8 weeks. At 5 months follow-up, the CT scans show integration of the bone graft both distally and proximally (Figure 7A and B), and the patent was allowed to bear full weight in a normal shoe.

He returned to a full-time posistion in a physically demanding job 10 months after surgery and was also able to take part in deer hunting at that time. Radiographs at 16 months demonstrated integration of the bone graft (Figure 7C and D). At final follow-up at 22 months, VAS score for pain at rest, and at walking was 1. The AOFAS midfoot scale score was 82, the SEFAS score was 39, and the MOxFQ score for walking/standing was 18, for pain 40, and for social interaction 6. He had little complains except reduced skin sensation dorso-medial and plantar of the great toe and soreness of the interphalangeal joint. The foot was well aligned (Figure 7E and F), and he walked without limping, when using shoes with custom made insoles.

## Discussion

We present 2 cases of a gunshot injury to the medial aspect of the foot with a defect of the first metatarsal bone. A structural iliac crest graft, internal fixation, and BMP-2 was used for reconstruction of the bony defect. In both cases, the structural bone graft was incorporated into the remaining viable proximal and distal bone. The patients were followed for almost 2 years, and they both returned to high-demand occupations and had good outcome scores.

The degree of tissue trauma following a gunshot depends on the tissues involved, the type of firearm, the projectiles physical characteristics, velocity, and distance of fire. Firearms are often classified into low and high velocity, or small- and large-arms weapons, respectively.^11^ Handguns, like the pistol as in case 1, are low-velocity weapons. Long-guns, like the rifle used for hunting in case 2, are high-velocity weapons.

In both cases, the gunshot resulted in multifragmentation of the MT1, well-circulated soft tissues and only minor loss of skin coverage. None of the patients experienced a wound infection or osteomyelitis following the reconstruction with bone graft and BMP-2. Debridement with repeated second-look surgeries was performed prior to reconstruction, and the patients received intravenous antibiotic. Intraoperative cultures were collected in case 2 at time of reconstruction but showed no bacteria. Husain et al published the outcome of 27 patients with gunshot injuries to the foot and ankle. When culturing the wounds before irrigation, the authors isolated bacteria in approximately 30% of the patients, but infection with osteomyelitis was only observed in one patient.^12^ Husain et al explained the low infection rate with administration of prophylactic antibiotics and wound management.

Various treatment options for bony defects of the femur and the tibia are described.^1-10,13-19^ Graft resorption can be a problem with conventional bone graft techniques, even when the area of injury is well-vascularized.^20^ When using the Masquelet technique a membrane induced by a cement spacer prevents graft resorption and favors its vascularity and its corticalization.^18^

The foot has lesser soft tissue coverage than femoral defects. The goal when reconstructing the defects following a gunshot injury must be soft tissue coverage, normal length of the rays and stability when possible. Gunshot wounds to the lower extremities are normally not life-threatening injuries and the need for amputations depends on soft tissue injury, bone injury, contamination, and the neurovascular situation.^21^ In many cases of gunshot injuries to the foot, large defects and compromised circulation are present. This leave no other choice than to complete a traumatic amputation or to perform a partial amputation of the injured foot. In other cases with extensive structural bone loss and significant injured soft tissues, a reconstruction of bony defects and soft tissue coverage can be achieved at simultaneously using osteocutaneous flaps from the fibula^7-9^ or radius.^22^

The literature regarding reconstruction of foot bone defects, is limited, and includes case series and case reports.^3,4,6-10^ An editorial from 2020, reviewing 41 studies, including 677 patients, found only 7 cases of metatarsal defects treated with the Masquelet technique.^23^ Only one paper describe iliac bone grafting, but the authors focus on soft tissue covering and not the use of a structural iliac crest graft.^5^ In case 1, we combined the Masquelet technique with a structural graft and BMP-2. A Cochrane review from 2010 evaluating the data from randomized controlled trials (RCTs) suggest that acute fracture patients treated with BMP required fewer secondary procedures compared to control groups.^16^ Studies, however, fail to provide definitive conclusions and recommendations regarding the use of BMP-2 for bone healing in fractures.^24-28^ The use of BMP-2 in these patients could be questioned, both because the disputed effect as well the high costs. In case 1, we implanted the cement spacer 10 days after the injury to create space for the next step surgery and a biologic membrane for increased healing. The BMP-2 was used to optimize healing. As the final reconstruction in case 2 was performed 15 weeks after injury, we chose direct graft implementation in combination with BMP-2, and no space- and biologic membrane creating procedures. The structural iliac crest graft was successfully integrated with the first metatarsal bone proximally, and the proximal hallux/metatarsal head distally at 3 to 5 months after graft implantation. Both patients reported donor site pain that lasted for some weeks. Iliac crest bone grafting is described to be a relative benign procedure with the most significant morbidity being pain.^29,30^ Both patients were able to return to physically demanding jobs, and both were walking without a limp. However, as shown on AOFAS midfoot scale, MOxFQ, EFAS scores, and the VAS score for pain, both patients had some minor complains at follow-up, and both patients were in the need for comfort footwear and/or shoe inserts.

## Conclusion

Gunshot injury with defect of the first metatarsal bone can be treated successfully with an iliac crest structural graft, internal fixation and BMP-2.

## Supplemental Material

sj-docx-1-fas-10.1177_19386400241278026 – Supplemental material for Gunshot Injury With Bone Defect of the First Metatarsal Bone A Presentation of 2 Cases Treated With an Iliac Crest Structural Graft, Internal Fixation, and Bone Morphogenic Protein 2 Supplemental material, sj-docx-1-fas-10.1177_19386400241278026 for Gunshot Injury With Bone Defect of the First Metatarsal Bone A Presentation of 2 Cases Treated With an Iliac Crest Structural Graft, Internal Fixation, and Bone Morphogenic Protein 2 by Elisabeth Ellingsen Husebye, Geir Stray Andreassen and Are Hauk00E5en St00F8dle in Foot & Ankle Specialist

sj-pdf-1-fas-10.1177_19386400241278026 – Supplemental material for Gunshot Injury With Bone Defect of the First Metatarsal Bone A Presentation of 2 Cases Treated With an Iliac Crest Structural Graft, Internal Fixation, and Bone Morphogenic Protein 2 Supplemental material, sj-pdf-1-fas-10.1177_19386400241278026 for Gunshot Injury With Bone Defect of the First Metatarsal Bone A Presentation of 2 Cases Treated With an Iliac Crest Structural Graft, Internal Fixation, and Bone Morphogenic Protein 2 by Elisabeth Ellingsen Husebye, Geir Stray Andreassen and Are Hauk00E5en St00F8dle in Foot & Ankle Specialist

sj-pdf-2-fas-10.1177_19386400241278026 – Supplemental material for Gunshot Injury With Bone Defect of the First Metatarsal Bone A Presentation of 2 Cases Treated With an Iliac Crest Structural Graft, Internal Fixation, and Bone Morphogenic Protein 2 Supplemental material, sj-pdf-2-fas-10.1177_19386400241278026 for Gunshot Injury With Bone Defect of the First Metatarsal Bone A Presentation of 2 Cases Treated With an Iliac Crest Structural Graft, Internal Fixation, and Bone Morphogenic Protein 2 by Elisabeth Ellingsen Husebye, Geir Stray Andreassen and Are Hauk00E5en St00F8dle in Foot & Ankle Specialist
