# Supplementary material for: Gunshot Injury With Bone Defect of the First Metatarsal Bone: A Presentation of 2 Cases Treated With an Iliac Crest Structural Graft, Internal Fixation, and Bone Morphogenic Protein 2
Source: Foot Ankle Spec. 2024 Sep 18;19(3):317–24. doi: 10.1177/19386400241278026 (PMC13144619; doi:10.1177/19386400241278026)
Supplement: sj-docx-1-fas-10.1177_19386400241278026 – Supplemental material for Gunshot Injury With Bone Defect of the First Metatarsal Bone A Presentation of 2 Cases Treated With an Iliac Crest Structural Graft, Internal Fixation, and Bone Morphogenic Protein 2 [file sj-docx-1-fas-10.1177_19386400241278026.docx]

**Captions to figures**

Figure 1. The figure shows the anteroposterior radiograph of the left foot demonstrating the projectile fragments and a shortened fragmented fiorst metatarsal (1A), the left foot with a gunshot injury with a debrided wound dorso medial and central plantar (1B and C). The foot is stabilized with an external fixator, and computed tomography scans of the injury demonstrating the metatarsal defect and metatarso phalangeal joint (1D, E and F).

Figure 2. The cement spacer was prepared (2A), and installed into the bony defect (2B). The radiograph shows the cement spacer in the region of the first metatarsal bone (2C).

Figure 3. An iliac crest bone graft was harvested from the ipsilateral side (3A), adjusted (3B) and installed into the defect (3C), stabilized with a plate, and finally covered with a sponge soaked with BMP2 (3D). The perioperative radiograph demonstrates the bone graft in the first metatarsal bone prior to plate fixation (3E).

Figure 4. The figure shows the postoperative computed tomography scan, with the bone graft and the metal plate for fixation (4A and B), the lateral and anterioposterior radiographs show the left foot one year after iliac graft implantation with integration of the graft (4C and D, the left foot from medial view (4E), and the left and right foot from the anterior view (4F) 23 months after surgery.

Figure 5. The initial anterioposterior and oblique radiographs show a multifragmented first metatarsal bone, fractured hallux sesamoids and proximal first phalanx (5A and B), and the computed tomography scans show the preoperative state of the injured right foot, with a defect of the first metatarsal bone including the metatarsal head (5C and D).

Figure 6. The figure shows the reaming of the graft (6A), the temporary fixation of the graft (6B and C), the stabilization with a plate (6D), and the sponge soaked with BMP-2 (6E).

Figure 7. The figure shows the anteroposterior and lateral computed tomographic scans of the right foot 5 months after the graft implantation. Both the distal and the proximal part of the graft seems integrated with the proximal phalanx and the proximal end of the first metatarsal bone, even though the central part of the graft shows hyperdensity (7A and B). The anteroposterior and lateral radiographs at 16 months after graft implantation show a continuity of the first metatarsus and a fused metatarsophalangeal joint (7C and D). Figure 7E shows the anterior view of the right and left foot and figure 7F the medial view of the right foot at 16 months after surgery.
