# Supplementary material for: Gunshot Injury With Bone Defect of the First Metatarsal Bone: A Presentation of 2 Cases Treated With an Iliac Crest Structural Graft, Internal Fixation, and Bone Morphogenic Protein 2
Source: Foot Ankle Spec. 2024 Sep 18;19(3):317–24. doi: 10.1177/19386400241278026 (PMC13144619; doi:10.1177/19386400241278026)
Supplement: sj-pdf-1-fas-10.1177_19386400241278026 – Supplemental material for Gunshot Injury With Bone Defect of the First Metatarsal Bone A Presentation of 2 Cases Treated With an Iliac Crest Structural Graft, Internal Fixation, and Bone Morphogenic Protein 2 [file sj-pdf-1-fas-10.1177_19386400241278026.pdf]

Oslo universitetssykehus

# Forespørsel om å kunne bruke informasjon om deg og din helse til publisering i tidsskrift eller i undervisning

*Vi ber om din tillatelse til å anvende helseopplysninger som er adekvate i forbindelse med skade i foten og behandling av denne skaden for publikasjon i ortopedisk medisinsk tidsskrift/undervisning av helsepersonell eller i forbindelse med konferanser. Dette inkluderer skadeårsak og omfang, bilder før/under og etter operasjon, rtg.bilder før og etter kirurgi, samt oppfølging etter operasjon.*

*Du vil ikke kunne gjenkjennes direkte i det som publiseres. Ditt navn og fødselsnummer vil ikke bli gjengitt. Likevel er det mulig at personer som kjenner din historie kan gjette at det er deg, og på den måten få vite mer om din skade.*

*Det er frivillig å akseptere at dine helseopplysninger anvendes for å belyse en problemstilling. Du kan når som helst før artikkel er sendt til tidsskrift for publisering, og uten å oppgi noen grunn, trekke ditt samtykke for anvendelse av dine helseopplysninger i case-beskrivelse planlagt for publikasjon. Dersom du trekker ditt samtykke, vil det ikke få konsekvenser for din videre behandling.*

*Dersom du ønsker det, kan du få se materialet når det er ferdig og før det skal brukes. Før case-beskrivelse ev. innsendes medisinsk tidsskrift, vil du få anledning til å gå igjennom case-beskrivelse for godkjennelse og ev. korrektur.*

*Du vil ikke ha noen spesielle fordeler av å samtykke, men informasjon om behandling av skaden vil senere kunne hjelpe andre ortopeder til å behandle pasienter med tilsvarende skade.*

*Dersom du samtykker til ovennevnte bes om at du underskriver samtykkeskjema*

Du har rett til innsyn i hvilke opplysninger som er lagret om deg og kan kreve at opplysningene blir rettet om opplysningene er feil.

**Kontaktinformasjon**
Dersom du ønsker mer informasjon, eller senere ønsker å trekke deg, kan du kontakte
Elisabeth Ellingsen Husebye, MD, PhD
+ 47 22118080
uxngng@ous-hf.no

Personvernombudet ved Oslo universitetssykehus kan bistå med generelle spørsmål knyttet til bruk og behandling av personopplysninger i forskning, samt kan kontaktes dersom du er usikker på om opplysningene behandles i tråd med hva som er beskrevet i samtykket: Telefon (+47) 915 02 770, eller på e-post personvern@ous-hf.no Se også www.oslo-universitetssykehus.no/personvern .

*Samtykke til publisering av kasuistikk*

# Samtykke

Jeg samtykker til at informasjon om meg og min helse kan brukes i artikler, som beskrevet i informasjonen ovenfor

Eirik Jansson, [illegible] 29.11.23

(Signert av pasient, dato)

# Bekreftelse på at informasjon er gitt pasienten

Jeg bekrefter å ha gitt informasjon som beskrevet ovenfor

Elisabeth Ell, [illegible] 10/11-23

(Signert, stilling, dato)
